# Aprepitant-loaded solid lipid nanoparticles: a novel approach to enhance oral bioavailability

**DOI:** 10.3762/bjnano.16.50

**Published:** 2025-05-15

**Authors:** Mazhar Hussain, Muhammad Farooq, Muhammad Asad Saeed, Muhammad Ijaz, Sherjeel Adnan, Zeeshan Masood, Muhammad Waqas, Wafa Ishaq, Nabeela Ameer

**Affiliations:** 1 Faculty of Pharmacy, The University of Lahore, Lahore, Pakistan; 2 School of Pharmacy, Multan University of Science and Technology, Multan, Pakistan; 3 Faculty of Pharmaceutical Sciences, University of Central Punjab, Lahore, Pakistan; 4 Department of Pharmacy, COMSATS University Islamabad, Lahore Campus, Lahore, Pakistan; 5 Faculty of Pharmacy, Grand Asian University Sialkot, Pakistan; 6 School of Engineering, Institute for Materials and Processes, The University of Edinburgh, Robert Stevenson Road, Edinburgh, EH9 3FB, United Kingdom

**Keywords:** aprepitant, β-cyclodextrin, pharmacokinetic study, poloxamer, solid lipid nanoparticles

## Abstract

Objectives of the present study are the development of aprepitant (APT)-loaded solid lipid nanoparticles (SLNs) using the polymers poloxamer 407 and β-cyclodextrin for enhanced solubility and their pharmacokinetic analysis. APT-loaded SLNs were prepared by the precipitation method and characterized by physicochemical studies including particle size and zeta potential measurements, drug content, encapsulation efficiency and solubility studies, Fourier-transform infrared spectroscopy (FTIR), scanning electron microscopy (SEM), X-ray diffraction (XRD), differential scanning calorimetry (DSC) and thermogravimetric analysis (TGA), in vitro drug release in 0.1 M HCl (pH 1.2) and phosphate-buffered saline (PBS, pH 7.4), and pharmacokinetic studies. The optimal formulation (APT-CD-NP4) containing the highest concentration of β-CD showed the highest drug solubility (93.50% ± 3.73%) in PBS (pH 7.4) and drug content (96.75% ± 0.24%); particle size, zeta potential, and polydispersity index of APT-CD-NP4 were 121.1 ± 0.72 nm, −18.8 ± 0.94 mV, and 0.15 ± 0.35, respectively. SEM analysis showed that APT was converted from the crystal state into an amorphous state after SLN preparation. FTIR results indicated compatibility between APT and the polymers. XRD, TGA, and DSC results indicated no physical interaction between drug and polymers. In vitro drug release studies showed that APT-CD-NP4 yielded the maximum drug release (98.89% ± 4.11%) in PBS (pH 7.4) and followed the Higuchi release model (with exponent *n* = 0.542), indicating non-Fickian diffusion (anomalous transport). The maximum concentration of drug in plasma and the bioavailability of optimal formulation APT-CD-NP4 were higher than those of pure APT. Therefore, the optimal SLN formulation APT-CD-NP4 is a promising tool for oral administration with sustained release to improve the bioavailability of the BCS class-IV drug APT.

## Introduction

Cancer is a major health problem worldwide, and cancer patients are treated by using conventional strategies such as surgery, chemotherapy, and radiotherapy, alone or in combination [1]. Chemotherapy is the basis of pharmacological cancer treatments [2]. The most common adverse effects in cancer patients induced by chemotherapy are nausea and vomiting (chemotherapy-induced nausea and vomiting, CINV), which occurs in 60–80% of cancer patients with negative impacts on the quality of life and patients’ treatment outcomes [3].

About 79% of patients face postoperative nausea and vomiting (PONV), with 10–40% anticipatory nausea and/or vomiting in those who receive chemotherapy. Factors that affect the incidence and severity of CINV in patients are schedule, dosage, route of administration of chemotherapy, age, sex, and history of alcohol use [4].

CINV are treated by commonly used agents such as dopamine antagonists, 5HT3 antagonists, and corticosteroids. In the first cycle of chemotherapy, acute emesis is prevented by administering a selective 5-HT3 receptor antagonist (ondansetron) together with a corticosteroid (dexamethasone) in 70–80% of cancer patients on day 1. Vomiting and significant nausea still occur in 25–40% of patients in the delayed phase (days 2 to 5) in their first cycle of high-dose cisplatin [5].

Aprepitant (APT) is a selective antagonist of neurokinin-1 receptor that blocks the substance P emetic effect. NK-1 receptors occur in the gastrointestinal tract on vagal afferents and in the nucleus of the solitary tract in the brain. APT is equally effective in the prevention of pre/postoperative nausea and as rescue antiemetic, offering better control of vomiting after 24 and 48 h compared with conventional therapies [6]. APT has an oral bioavailability of 60–65%. The maximum concentration of drug in plasma (*C*_max_) is reached after approx. 4 h, and the half-life is 9–13 h. An oral dose of 125 mg APT one hour before chemotherapy treatment (day 1), and 80 mg daily in the morning on days 2 and 3 are recommended [7]. APT is strongly bound to plasma protein (95%); it is absorbed slowly and crosses the blood–brain barrier [8]. Its mean volume of distribution is approximately 70 L. In the range of pH 2–10, APT has very low solubility (0.37 µg/mL) [9].

Because of the low water-solubility, the low permeability, and the rate-limiting step of poor gastrointestinal absorption, APT is categorized as a BCS class-IV drug [10]. Low solubility and poor dissolution of BCS class-IV drugs can be improved by using techniques such as incorporating the drug or prodrug into lipid or polymeric formulations, using solid lipid nanoparticles (SLNs), applying surfactants, adjusting the pH value, reducing particle size, or applying cyclodextrin complexation or salt formation. However, these techniques depend on the physicochemical features of the drug in each case. SLNs are the easiest and most suitable scalable of the above approaches for developing stable commercially viable dosage forms [11].

Various methods such as hot homogenization, cold homogenization, and solvent evaporation are used to prepare SLNs, but precipitation is the most compatible method for compound, polymer, and lipid. The precipitation method consists of dissolving the lipid with organic solvents and adding water to cause supersaturation of solid lipids in the mixture, which leads to precipitation of SLNs [12]. The precipitation method has the advantage of simple, rapid, reproducible, cost-effective, and easy handling for producing SLNs with good scale-up potential [13].

In the present study, we prepared SLNs containing APT with better solubility and enhanced dissolution using a minimum quantity of carriers. The developed SLNs were evaluated regarding drug content and using scanning electron microscopy (SEM), thermal gravimetric analysis (TGA) and differential scanning calorimetry (DSC), as well as polydispersity index (PDI), particle size, and zeta potential measurements. Also Fourier-transform infrared (FTIR) spectroscopy, X-ray diffraction (XRD), solubility, in vitro dissolution, and in vivo and stability studies were carried out.

## Result and Discussion

### Physicochemical evaluation

**Figure 1:**
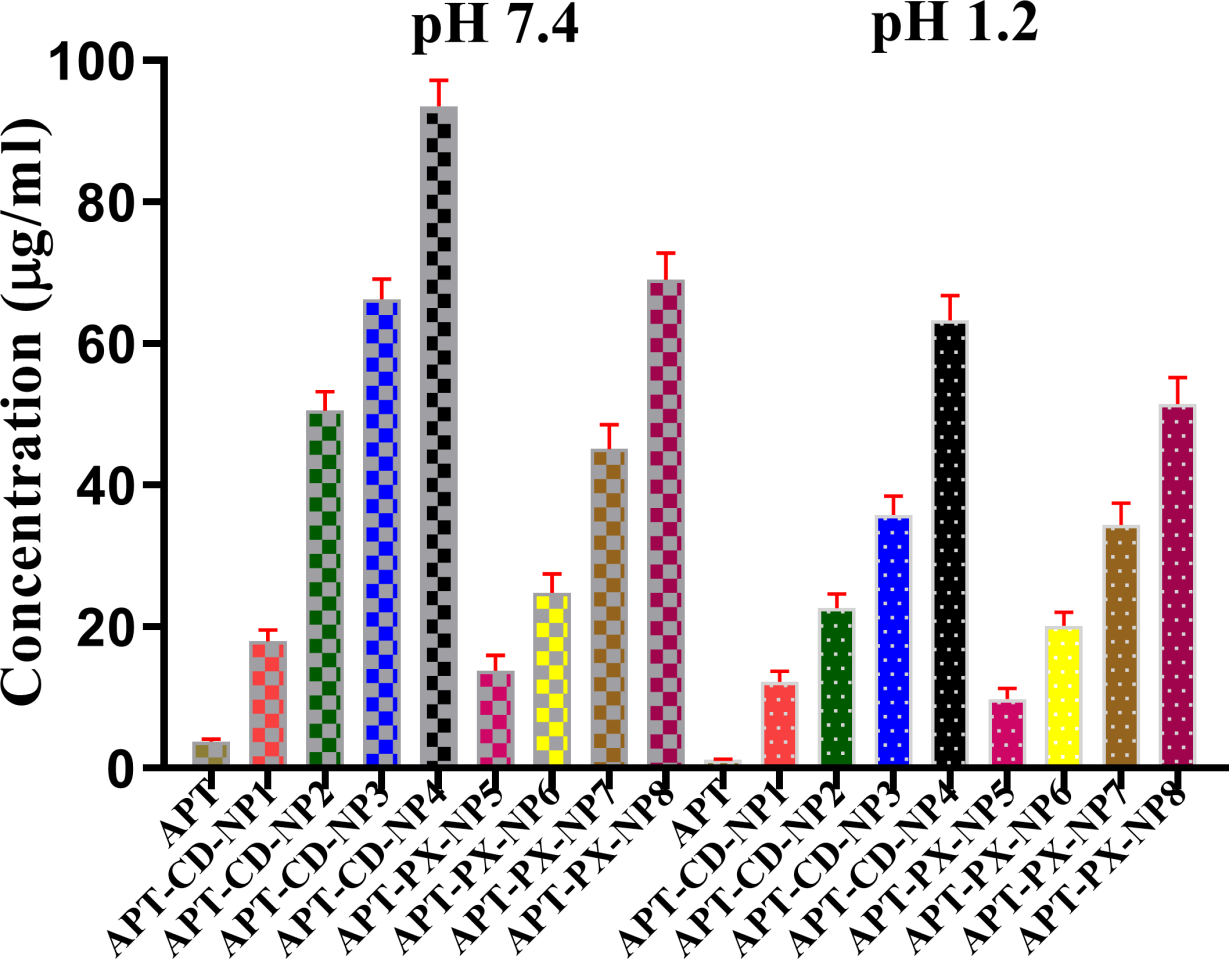
Solubility analysis of APT and APT-loaded SLNs in 0.1 N HCl (pH 1.2) and phosphate-buffered saline (pH 7.4).

The solubility of APT in the SLNs was 24-fold higher than that of APT in PBS (pH 7.4) and acidic medium (0.1 M HCl, pH 1.2). The samples APT-CD-NP1 to APT-CD-NP4 contained β-cyclodextrin (β-CD), while samples APT-PX-NP5 to APT-PX-NP8 contained poloxamer 407, both in different proportions (Figure 1). The order of solubility was APT-CD-NP1 > APT-CD-NP2 > APT-CD-NP3 > APT-CD-NP4 due to the gradual increase of β-CD, and the optimum solubility was achieved with APT-CD-NP4 in both solvents.

Better solubility was achieved in PBS (pH 7.4) compared to acidic medium due to the lipophilic nature of APT loaded into the interior cavity of β-CD [11]. An increased β-CD concentration converted crystalline entrapped APT into an amorphous state, decreasing surface tension and promoting the solubility profile [14]. A similar behavior was observed for the poloxamer 407 samples with solubilities in the order of APT-PX-NP5 > APT-PX-NP6 > APT-PX-NP7 > APT-PX-NP8 due to the gradual increase in poloxamer 407 concentration. APT-PX-NP5 to APT-PX-NP8 exhibited a 4 to 18 times higher solubility in PBS (pH 7.4) and acidic medium compared to pure APT because of the presence of poloxamer 407, which is an amphiphilic triblock copolymer of poly(propylene oxide) (PPO) and poly(ethylene oxide) (PEO). The PPO segments are hydrophobic, while the PEO segments are hydrophilic [15]. Comparing APT-CD-NPs and APT-PX-NPs, the solubility in APT-CD-NPs was higher because β-CD is more hydrophilic than poloxamer 407. Poloxamer 407 shows thermoreversible properties.

### Drug content and encapsulation efficiency

The drug content of SLNs formulations ranged from 16.95% ± 0.76% to 96.75% ± 0.24%; the highest APT content was obtained in APT-CD-NP4. The loss of the drug can be attributed to the lyophilization. However, there was no change in color or aggregation observed. Dispersions of lyophilized SLN formulations in distilled water exhibited a homogeneous white color. The encapsulation efficacy of the SLNs formulations was in the range of 25.33% ± 0.89% to 80.55% ± 0.15%. A higher content of β-CD in the formulation enhanced the encapsulated amount of APT. The hydrophobic structure of β-CD facilitates strong interaction with APT, resulting the enhanced encapsulation efficiency. Nazli Erdogar et al. achieved a higher encapsulation efficiency for aprepitant with PEG–chitosan-coated cyclodextrin nanocapsules [16].

#### Zeta potential, particle size analysis and polydispersity index

Zeta potential is a key factor in evaluating stability of APT-loaded SLNs. The zeta potential values of the prepared SLNs (APT-CD-NP1 to APT-PX-NP8) were −25.7 ± 0.21, −24.2 ± 0.31, −20.8 ± 0.41, −18.8 ± 0.94, −23.5 ± 0.75, −22.8 ± 0.64, −22.7 ± 0.52 and −20.7 ± 0.71 mV, respectively (Table 1). Among all formulations, APT-CD-NP4 and APT-PX-NP8 were the most stable. The presence of free fatty acids causes a high negative charge on the prepared formulations. A zeta potential of ±30 mV is sufficient to form stable particle systems [17].

**Table 1:** Drug content, encapsulation efficiency, particle size, zeta potential, and polydispersity index (PDI) of SLN formulations before and after redispersion.

Formulation	Drug content (%)	Encapsulation efficiency (%)	Particle size (nm)		Zeta potential (mv)		PDI	
			before redispersion	after redispersion	before redispersion	after redispersion	before redispersion	after redispersion

APT−CD−NP1	21.52 ± 0.43	33.16 ± 0.88	204.9 ± 0.3	200.0 ± 0.1	−25.7 ± 0.2	−25.0 ± 0.1	1.0 ± 0.2	1.0 ± 0.4
APT−CD−NP2	39.76 ± 0.32	40.17 ± 0.23	168.3 ± 0.5	160.0 ± 0.6	−24.2 ± 0.3	−23.0 ± 0.2	1.0 ± 0.4	1.0 ± 0.5
APT−CD−NP3	68.40 ± 0.55	55.44 ± 0.58	141.3 ± 0.6	139.0 ± 0.8	−20.8 ± 0.4	−20.0 ± 0.4	1.0 ± 0.2	1.0 ± 0.3
APT−CD−NP4	96.75 ± 0.24	80.55 ± 0.15	121.1 ± 0.7	116.0 ± 0.2	−18.8 ± 0.9	−17.0 ± 0.6	0.2 ± 0.4	0.1 ± 0.5
APT−PX−NP5	16.95 ± 0.76	25.33 ± 0.89	257.6 ± 0.4	251.0 ± 0.7	−23.5 ± 0.8	−24.0 ± 0.5	1.0 ± 0.3	1.0 ± 0.6
APT−PX−NP6	31.00 ± 0.43	39.45 ± 0.22	229.5 ± 0.9	225.0 ± 0.4	−22.8 ± 0.6	−22.0 ± 0.3	1.0 ± 0.5	1.0 ± 0.7
APT−PX−NP7	54.30 ± 0.82	58.10 ± 0.45	207.2 ± 0.6	205.0 ± 0.2	−22.7 ± 0.5	−22.0 ± 0.8	1.0 ± 0.3	1.0 ± 0.1
APT−PX−NP8	84.25 ± 0.36	77.52 ± 0.19	191.0 ± 0.6	190.0 ± 0.7	−20.7 ± 0.7	–20.0 ± 0.9	0.1 ± 0.5	0.1 ± 0.6

**Figure 2:**
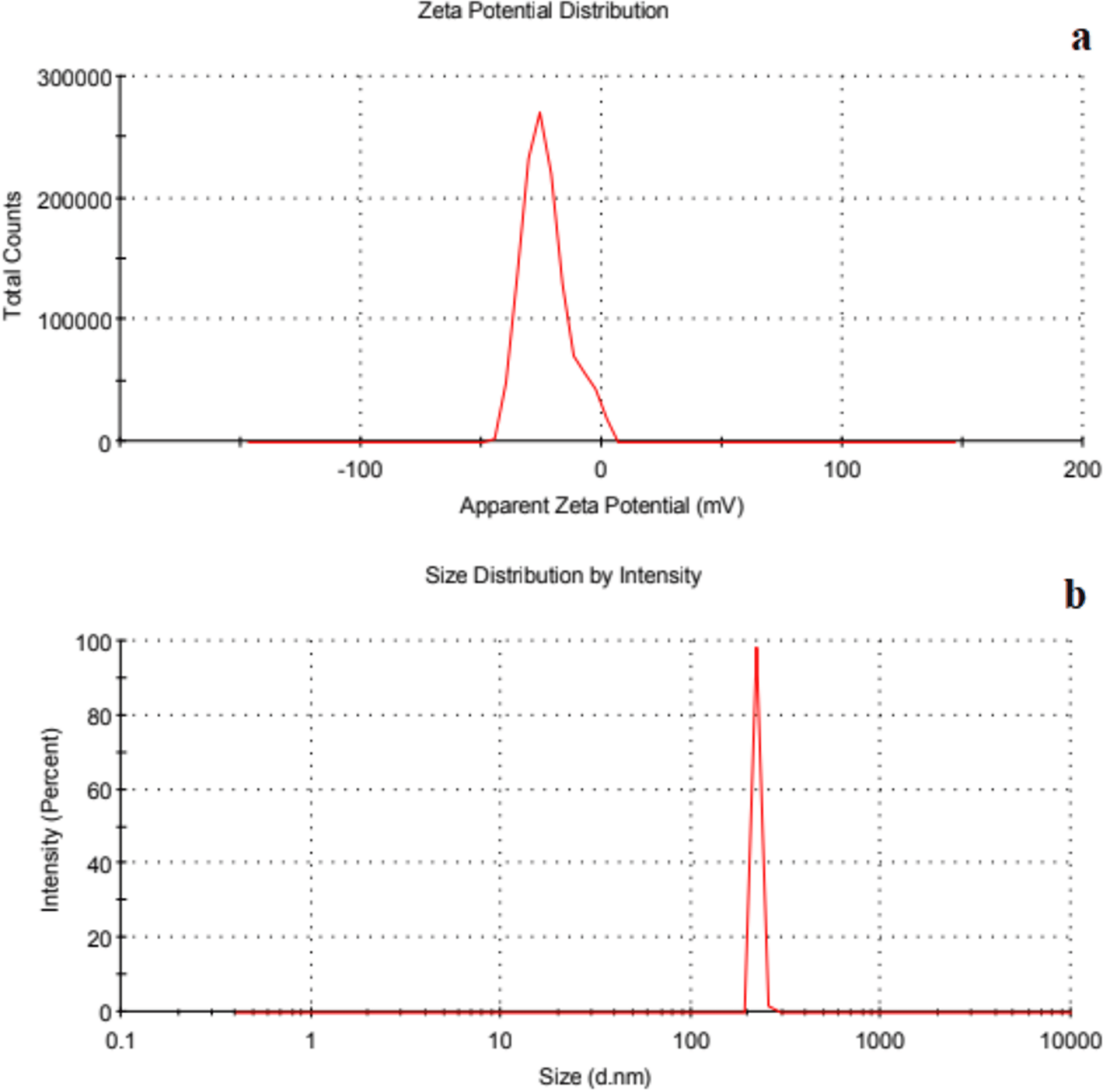
(a) Zeta Potential and (b) particle size of APT-CD-NP4.

The particle sizes of APT-CD-NP1 to APT-PX-NP8 were 204.9 ± 0.31, 168.3 ± 0.45, 141.3 ± 0.62, 121.1 ± 0.72, 257.6 ± 0.37, 229.5 ± 0.94, 207.2 ± 0.63, and 191.0 ± 0.57 nm, respectively (Table 1). An exemplary measurement for APT-CD-NP4 is given in Figure 2. The SLNs with lower particle size provide a large surface area, which increases drug release and enhances drug absorption by reducing the thickness of the diffusional layer in the gastrointestinal tract. The PDI is a parameter used to define particle size distribution. PDI values above 0.7 indicate a disperse particle size distribution. The APT-loaded SLNs APT-CD-NP4 and APT-PX-NP8 showed PDI values below 0.2, indicating uniform size dispersity. Table 1 shows that when the β-CD concentration was reduced, particle size and PDI increased and the zeta potential value changed to more negative values. The results indicate that APT-loaded SLNs effectively redisperse into stable NPs while maintaining their original physicochemical properties. The zeta potential values of all formulations remained negative, demonstrating that the electrostatic stability of SLNs was maintained. APT-CD-NP4 (from −18.8 ± 0.94 to −17 ± 0.6 mV) and APT-PX-NP8 (from −20.7 ± 0.71 to −20.0 ± 0.9 mV) showed only slight shifts. The particle size of all formulations remained almost constant after redispersion, with only minor variations observed. APT-CD-NP1 showed a slight reduction from 204.9 ± 0.3 to 200.0 ± 0.1 nm, and the size of APT-PX-NP5 decreased from 257.6 ± 0.3 to 251.0 ± 0.7 nm. These small changes indicated efficient redispersion without significant particles aggregation. The PDI values remained stable across all formulations after redispersion. APT-CD-NP4 (from 0.2 ± 0.4 to 0.1 ± 0.5) and APT-PX-NP8 (0.1 ± 0.5 to 0.1 ± 0.6) retained their monodisperse distribution, while the PDI of other formulations remained at 1.0, indicating controlled dispersion.

#### SEM studies

**Figure 3:**
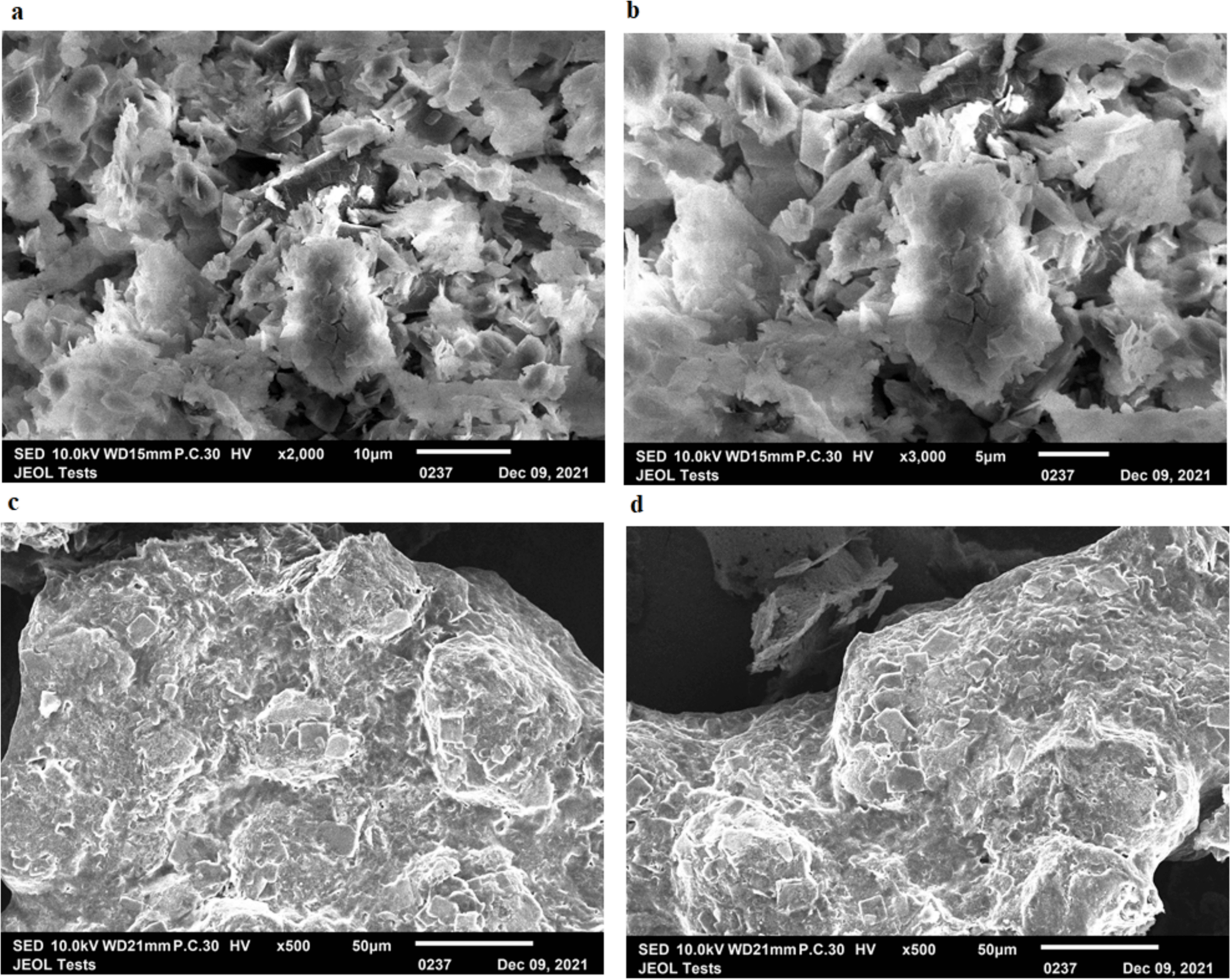
SEM photomicrographs of (a, b) APT-CD-NP4 and (c, d) APT-PX-NP8.

Scanning electron micrographs of APT-CD-NP4 and APT-PX-NP8 shown in Figure 3 illustrate that polymeric content was deposited on the SLN surface because of organic solvents. After evaporation of the organic solvent, colloidal particles are closely packed. Dispersions in organic solvents were stronger and minimized the formation of cracks. However, APT-loaded SLNs formulations in the aqueous phase appeared as nonspherical granules.

#### FTIR studies

**Figure 4:**
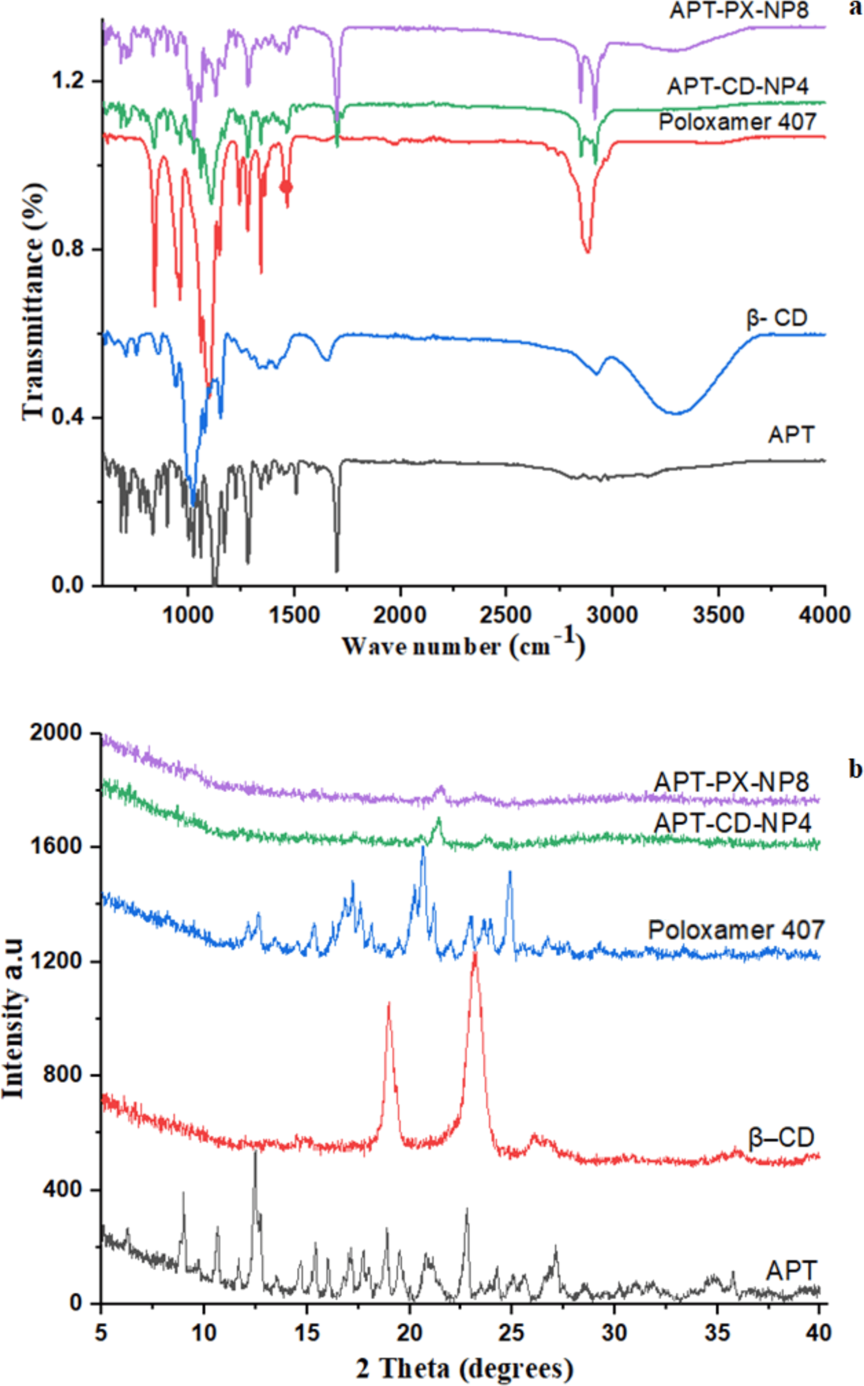
(a) FTIR spectra and (b) X-ray diffractograms of APT, poloxamer 407, β-CD, APT-CD-NP4, and APT-PX-NP8.

FTIR spectroscopy was applied to estimate any interaction between APT and β-CD or poloxamer 407 in APT-CD-NP4 and APT-PX-NP8. Figure 4a shows characteristic peaks of APT at 1119 and 1168 cm^−1^, corresponding to C–F stretching, while peaks at 1224 and 1279 cm^−1^ were attributed to C–O stretching. Other peaks were identified at 1510 cm^−1^ (C=C stretching) and 1699 cm^−1^ (C=O stretching) [18]. The characteristic peaks of poloxamer 407 were at 841 cm^−1^ (O–H group) and 1102 cm^−1^ (C–O stretching), 1341 cm^−1^ (O–H bending), and 2884 cm^−1^ (C–H stretching aliphatic) (Figure 4a). The characteristic peaks of β-CD were at 1020 cm^−1^ (C–O–C symmetric stretching), 1154 cm^−1^ (C–O–C asymmetric stretching), 1660 cm^−1^ (H–O–H bond deformation of water), 2931 cm^−1^ (C–H stretching), and 3316 cm^−1^ (OH group stretching) (Figure 4a).

The formulated SLNs APT-CD-NP4 and APT-PX-NP8 displayed prominent peaks at 1110 cm^−1^ (C–F stretching), 1168 cm^−1^ (C–F stretching), 1224 cm^−1^ (C–O stretching), 1279 cm^−1^ (C–O stretching), 1510 cm^−1^ (C=C stretching), and 1699 cm^−1^ (C=O stretching). The peak at 1119 cm^−1^ slightly shifted to 1110 cm^−1^ in APT-loaded SLNs formulations (Figure 4a). The peaks at 2849 and 2921 cm^−1^ are attributed to C–H bonds of poloxamer 407 or β-CD. A reduction in intensity and shift of the polymer peaks indicates the interaction between APT and polymers [19].

#### X-ray diffraction studies

APT, β-CD, poloxamer 407, APT-CD-NP4, and APT-PX-NP8 were evaluated using X-ray diffraction. APT exhibited sharp and intense peaks at diffraction angles (2θ) of 8.98°, 10.64°, 12.47°, 14.67°, 15.42°, 17.13°, 18.89°, 19.48°, 20.81°, 22.78°, and 27.10° (Figure 4b). The XRD pattern of β-CD showed peaks at 19.00° and 23.24°, and poloxamer 407 showed peaks at 12.66°, 15.37°, 17.23°, 20.64°, 21.19°, 23.69°, and 24.89°. The XRD patterns of APT-CD-NP4 and APT-PX-NP8 showed intense peaks at 21.56° and 21.37°, respectively (Figure 4b). The drug-loaded SLNs formulations exhibited less sharp peaks than APT because of a reduction of the polymer crystallinity. Also, the interaction of polymeric content with APT via hydrogen bonding converts the crystalline form of APT into an amorphous form [20].

#### TGA and DSC studies

**Figure 5:**
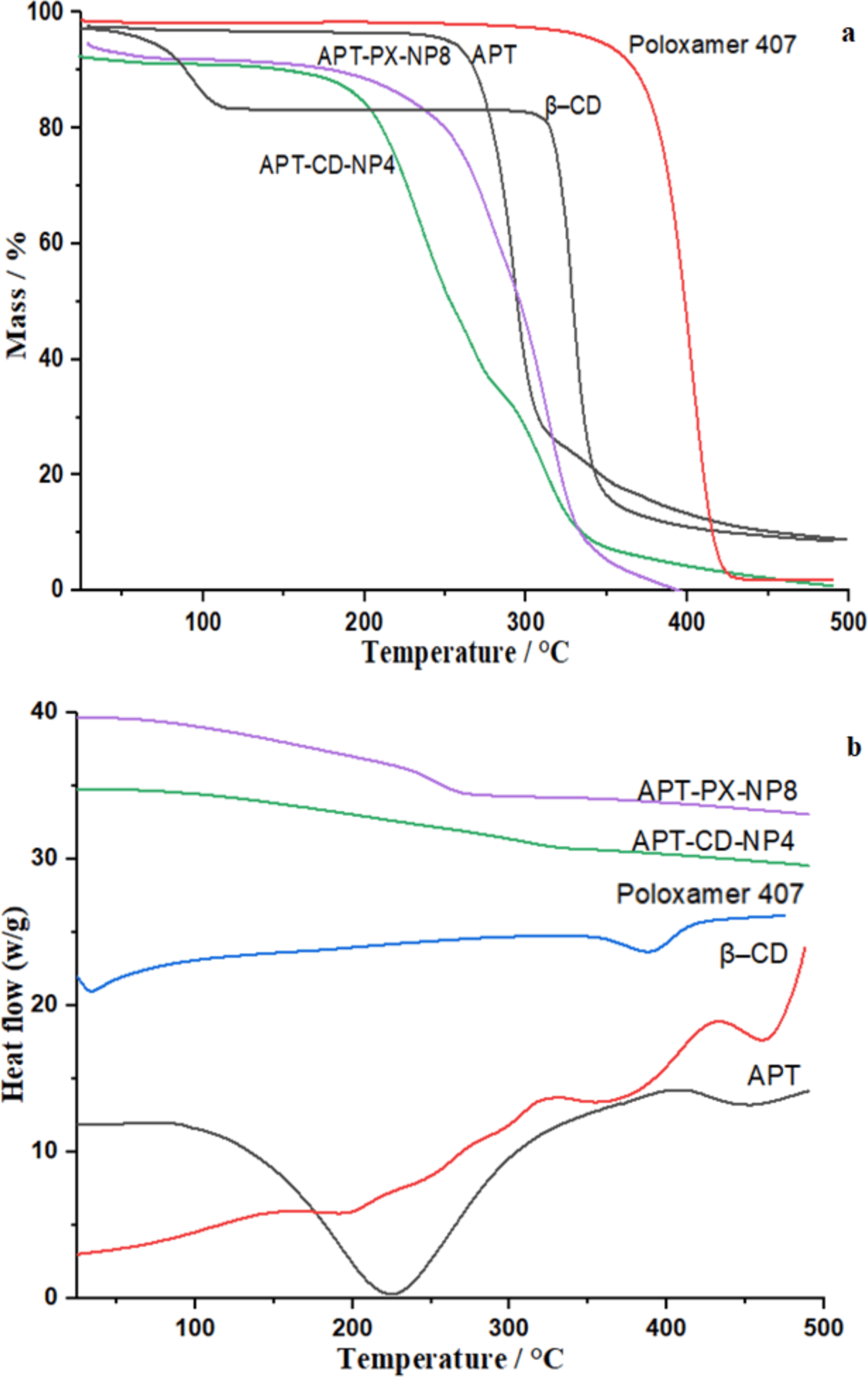
(a) TGA and (b) DSC thermograms of APT, poloxamer 407, β- CD, APT-CD-NP4 and APT-PX-NP8.

Thermogravimetric analysis showed that the thermal decomposition of APT occurred in two steps. The first step from 230 to 315 °C corresponded to the elimination of physically absorbed water, and in a second step, further weight loss was observed at 315 to 500 °C, corresponding to the formation of volatile molecules through oxidation (Figure 5a). β-CD and poloxamer 407 appeared to be more stable with mass losses of 80% and 70%, respectively, due to thermal decomposition between 330 and 480 °C. APT-CD-NP4 and APT-PX-NP8 weight losses of more than 80% were observed between 145 and 330 °C. For, APT-PX-NP8, there was complete weight loss at 430 °C.

DSC tests (Figure 5b) were conducted to the study physical state of APT, β-CD, poloxamer 407, and APT-loaded SLNs formulations. APT exhibits an endothermic melting peak at 255 °C, which indicates a phase transition of APT. An endothermic peak of β-CD is seen at 100 °C, which is associated with the release of water from β-CD; the endothermic peak at 330 °C corresponds to the beginning decomposition of β-CD. The endothermic peak of poloxamer 407 at 48 °C corresponds to its melting, and another broad peak observed at 400 °C can be attributed to the thermal decomposition of poloxamer 407. APT-CD-NP4 showed a wide peak at 100 to 300 °C, and APT-PX-NP8 showed a peak at 310 °C (Figure 5b). The melting peaks of APT disappeared in the SLNs because of the molecular encapsulation of APT in the polymeric cavity. This indicates a strong interaction between polymers and APT [21]. The DSC results are in line with XRD and TGA results, which show that APT in the APT-loaded SLNs is amorphous.

#### In vitro drug release studies

**Figure 6:**
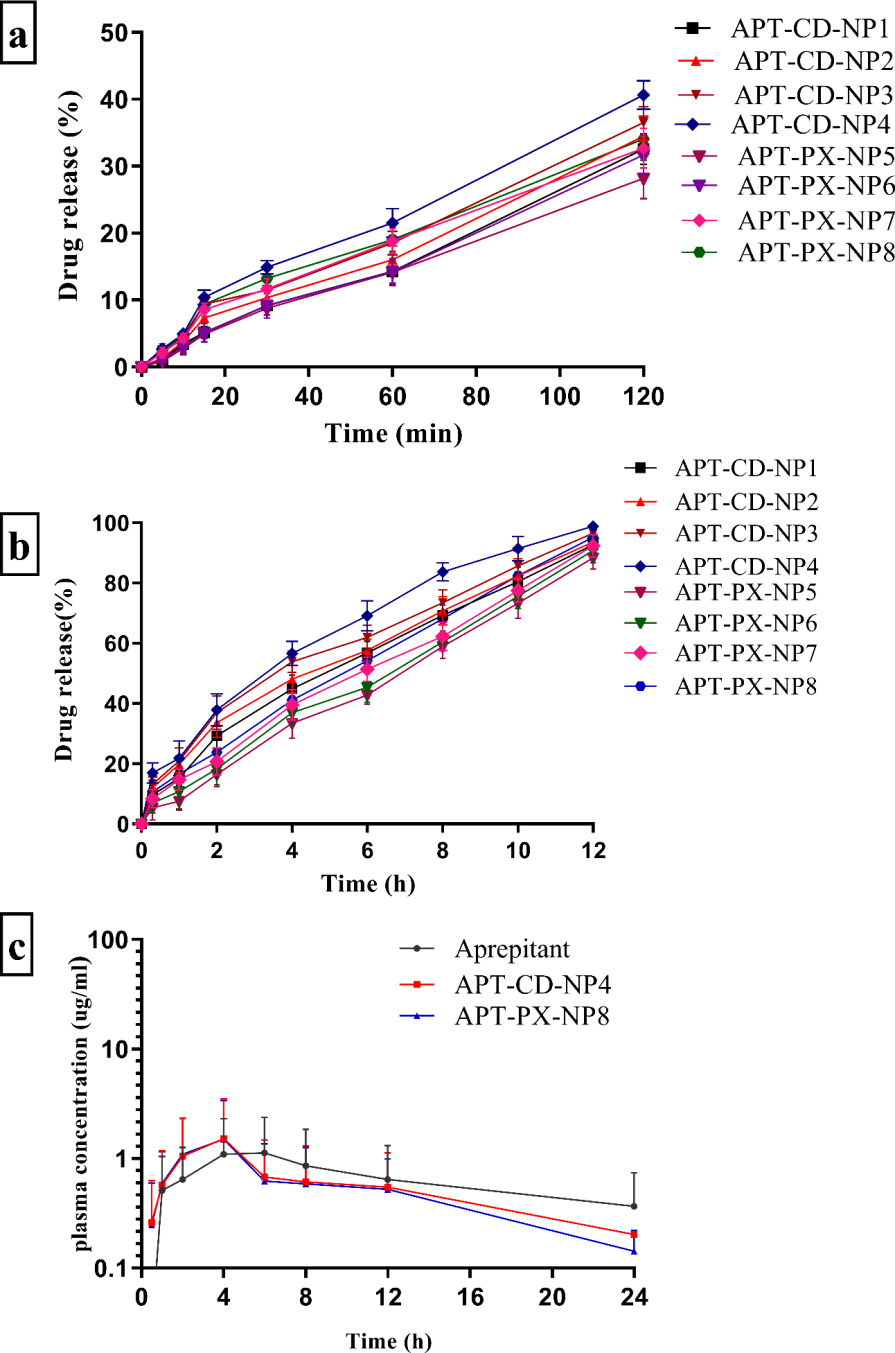
In vitro release of APT from SLN formulations in (a) 0.1 M HCl (pH 1.2) and (b) phosphate-buffered saline (pH 7.4). (c) Plasma concentration of APT, APT-CD-NP4, and APT-PX-NP8.

In vitro drug release studies (Figure 6) were carried out to study the effect of β-CD and poloxamer 407 on APT release. Results showed 40.65% ± 2.15% release of APT from APT-CD-NP4; APT-PX-NP8 released 34.05% ± 4.17% within 2 h in acidic medium containing 0.1 M HCl (pH 1.2); the drug release rates in PBS (pH 7.4) within 12 h were 98.89% ± 4.11% and 95.19% ± 4.53%, respectively. APT release from APT-CD-NP4 was higher than from APT-PX-NP8. Erdogar et al. showed a similar pattern of APT release from PEG/chitosan-coated cyclodextrin nanocapsules in acidic and basic media [16]. There is increased wetting of amorphous APT after loading into SLNs with increased surface area leading to rapid and consistent release.

#### Drug release kinetics

The drug release data obtained from APT-CD-NP1 to APT-PX-NP8 were analyzed using various kinetic models, that is, zeroth-order, first-order, Higuchi, and Korsemyer–Peppas models by applying Microsoft Excel (DD Solver). Table 2 shows that APT-CD-NP1, APT-CD-NP2, APT-CD-NP3, APT-CD-NP4, APT-PX-NP5, and APT-PX-NP6 followed the zeroth-order model in acidic medium (pH 1.2) with *R*^2^ values of 0.9133, 0.9829, 0.988, 0.9874, 0.9969, and 0.9924, respectively, while APT-PX-NP7 and APT-PX-NP8 followed concentration-dependent first-order release with *R*^2^ values 0.9769 and 0.9663, respectively. In PBS (pH 7.4), APT-PX-NP5, APT-PX-NP6, and APT-PX-NP7 followed zeroth-order release with *R*^2^ values of 0.9962, 0.9901, and 0.9771, respectively (Table 3). APT-CD-NP1 and APT-PX-NP8 follow the first-order model with *R*^2^ values of 0.9859 and 0.9719, while APT-CD-NP2, APT-CD-NP3, and APT-CD-NP4 followed the Higuchi model with *R*^2^ values 0.9876, 0.9923, and 0.9936, showing that the drug release is controlled through diffusion and changes over time. In the Korsemyer–Peppas model, the values of the exponent of drug release (*n*) of APT-CD-NP1 to APT-PX-NP8 in acidic medium ranged from 0.723–0.978 with regression coefficients *R*^2^ = 0.9870–0.9962, indicating anomalous APT release with non-Fickian diffusion (Table 2).

**Table 2:** Results of kinetic modeling of SLNs formulations in 0.1 N HCl (pH 1.2).

Formulation	Zeroth order (*R*^2^)	First order (*R*^2^)	Higuchi (*R*^2^)	Korsemyer–Peppas	
				(*R*^2^)	n

APT-CD-NP1	0.9913	0.9864	0.8396	0.9914	0.978
APT-CD-NP2	0.9829	0.9824	0.8678	0.9880	0.892
APT-CD-NP3	0.9888	0.9794	0.8971	0.9870	0.812
APT-CD-NP4	0.9874	0.9780	0.9165	0.9895	0.765
APT-PX-NP5	0.9969	0.9952	0.8677	0.9962	0.903
APT-PX-NP6	0.9924	0.9888	0.8421	0.9927	0.971
APT-PX-NP7	0.9549	0.9769	0.9269	0.9942	0.748
APT-PX-NP8	0.9407	0.9663	0.9329	0.9902	0.723

**Table 3:** Results of kinetic modeling of SLNs formulations in phosphate-buffered saline (pH 7.4).

Formulation	Zeroth order (*R*^2^)	First order (*R*^2^)	Higuchi (*R*^2^)	Korsemyer–Peppas	
				(*R*^2^)	n

APT-CD-NP1	0.9420	0.9859	0.9738	0.9983	0.666
APT-CD-NP2	0.9033	0.9778	0.9876	0.9972	0.596
APT-CD-NP3	0.8727	0.9772	0.9923	0.9963	0.560
APT-CD-NP4	0.8524	0.9821	0.9936	0.9957	0.542
APT-PX-NP5	0.9962	0.9633	0.8974	0.9972	0.943
APT-PX-NP6	0.9901	0.9658	0.9166	0.9955	0.873
APT-PX-NP7	0.9771	0.9730	0.9421	0.9957	0.782
APT-PX-NP8	0.9681	0.9719	0.9538	0.9964	0.741

Regarding the measurements in PBS (pH 7.4), the value of *n* of all formulations APT-CD-NP1 to APT-PX-NP8 ranged from 0.542 to 0.943, demonstrating non-Fickian diffusion with anomalous drug transport. The drug release from polymer-based APT-loaded formulations is as follows: When the polymeric membrane comes in contact with the aqueous medium, it absorbs water and swells. Water penetrates the APT-loaded SLN formulations fast and passes through toward the drug core such that the drug dissolves. Diffusion is enhanced because of the equilibrium between elastic polymer strength and hydration by increasing the swelling of the polymer [22]. In the dissolution medium, pores are formed in the polymeric membrane that determine the release of the drug through osmotic pressure difference [16].

#### Statistical analysis

The drug release from the formulated SLNs was analyzed statistically using ANOVA, followed by post hoc Dunnett’s test to investigate differences in APT release from all formulations. All formulations were compared to APT-CD-NP4. Results showed that the difference to APT-CD-NP3 is non-significant and the one to APT-CD-NP2 is of little significance (*P* < 0.0005). However, the differences to APT-CD-NP1, APT-CD-NP2, APT-PX-NP5, APT-PX-NP6, APT-PX-NP7, and APT-PX-NP8 were significant (*P* < 0.0001) as shown in Table 4. Cumulative drug release in APT-CD-NP4 was 98.89%, while in the other formulations it was up to 95.19%. The differences between formulations concern amount and type of polymer.

**Table 4:** Multiple Dunnett’s test on in vitro drug release between SLN formulations.

Dunnett’s comparisons	Mean difference	95.00% confidence interval of difference	Significant	P

APT-CD-NP4 vs APT-CD-NP1	8.677	4.586 to 12.77	yes	<0.0001
APT-CD-NP4 vs APT-CD-NP2	6.497	2.406 to 10.59	yes	0.0005
APT-CD-NP4 vs APT-CD-NP3	3.728	−0.3631 to 7.819	no	0.0879
APT-CD-NP4 vs APT-PX-NP5	16.69	12.60 to 20.78	yes	<0.0001
APT-CD-NP4 vs APT-PX-NP6	14.56	10.47 to 18.65	yes	<0.0001
APT-CD-NP4 vs APT-PX-NP7	12.15	8.056 to 16.24	yes	<0.0001
APT-CD-NP4 vs APT-PX-NP8	9.359	5.268 to 13.45	yes	<0.0001

#### Stability studies

**Figure 7:**
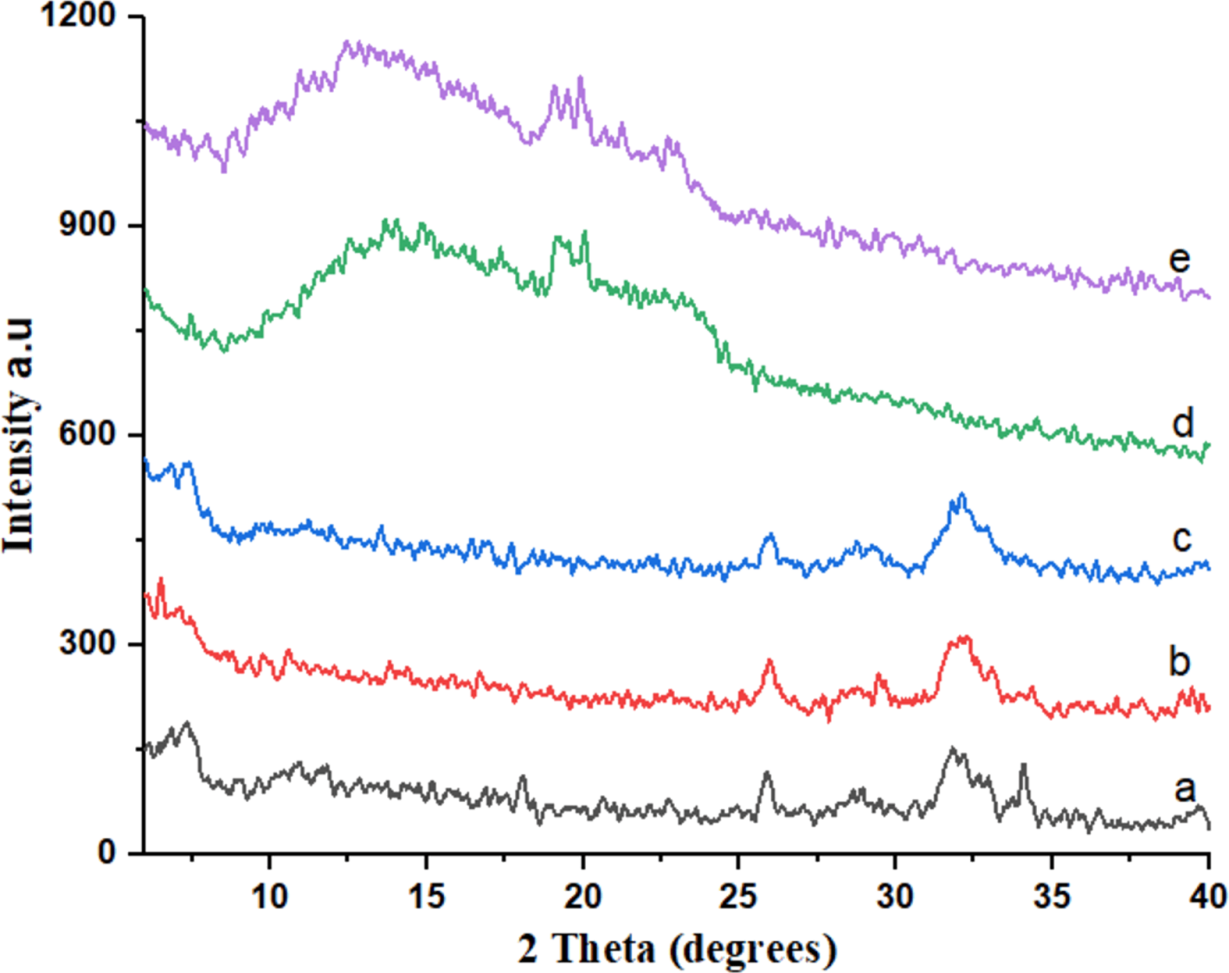
X-ray diffractograms of the optimal formulation APT-CD-NP4 after the accelerated stability studies. (a) Initial sample, (b) after one month, (c) after two months, (d) after three months, and (e) after six months.

For stability studies, the optimal sample APT-CD-NP4 was used. The samples were exposed to 40 °C/75% RH for 0, 1, 2, 3, and 6 months. Figure 7 shows the XRD patterns of the corresponding samples. Freshly prepared APT-CD-NP4 showed peaks at 2θ values of 6.95° (270), 7.4° (290), and 32.1° (244) (Figure 7a). After one and two months, peaks at 2θ values of 26.0° (227), 32.0° (257), and 31.8° (256) were measured (Figure 7b,c). After three and six months, peaks at 2θ values of 13.75° (446) and 13.0° (488) are seen (Figure 7d,e). Figure 7 shows that the peak intensity of APT in APT-CD-NP4 is lower than the peak intensity of pure APT during XRD analysis. Therefore, it was concluded that APT dispersed in a SLN was converted to a physically stable amorphous form and distributed uniformly. The results show that APT in the SLN remained amorphous after exposure to the high temperature and humidity in the long-term stability study.

#### In vivo pharmacokinetic studies

Pharmacoikinetic studies showed a linear relationship between APT release and concentration in rabbit blood plasma (*R*^2^ = 0.9959). The pharmacokinetic parameters determined by measuring plasma concentration versus time are displayed in Figure 6c and Table 5. The *C*_max_ values of groups treated with APT, APT-CD-NP4, and APT-PX-NP8 (equal to 5.5 mg/kg APT) were 2.0 ± 0.7, 2.9 ± 0.3, and 2.8 ± 0.4 μg/mL, respectively. The *C*_max_ values of SLN APT-CD-NP4 and APT-PX-NP8 were increased, respectively, 1.45 and 1.41 times compared to pure APT because of the surfactant in the formulations. APT-CD-NP4 showed a better outcome than APT-PX-NP8 because of the influence of β-CD. We propose that the medication has great penetrability in the gastrointestinal tract and would be quickly ingested to the same extent as to which it had been solubilized.

**Table 5:** Pharmacokinetic parameters of pure APT and APT-loaded formulations.^a^

Formulation	*t*_1/2_ (h)	*T*_max_ (h)	*C*_max_ (µg/mL)	(AUC)_0–_*_t_* (µg/mL·h)	(AUC)_0–∞_ (μg/mL·h)	(MRT)_0–∞_ (h)

APT	12.68 ± 0.14	6.0 ± 0.3	2.0 ± 0.7	27.7 ± 4.6	39.2 ± 3.33	19.57 ± 1.66
APT-CD-NP4	6.38 ± 0.19	4.0 ± 0.2	2.9 ± 0.3	24.1 ± 2.1	26.0 ± 1.6	9.83 ± 0.98
APT-PX-NP8	6.38 ± 0.14	4.0 ± 0.3	2.8 ± 0.4	23.3 ± 3.4	25.1 ± 2.2	9.58 ± 0.76

^a^*t*_1/2_: half-life; *T*_max_: time to reach the maximum drug concentration in plasma; *C*_max_: maximum drug concentration in plasma; AUC: area under the plasma concentration curve; MRT: mean residence time.

The *T*_max_ values of APT, APT-CD-NP4, and APT-PX-NP8 were 6.0 ± 0.3, 4.0 ± 0.2 and 4.0 ± 0.3 h, respectively. The *T*_max_ values of APT-CD-NP4 and APT-PX-NP8 were lower than that of APT; thus, absorption of APT from SLNs was faster, indicating that the dispersion of pure APT from the intestinal lumen through intestinal barrier was slower. Precipitation is another factor that could explain the slow absorption of pure APT [23]. The quick absorption of APT from SLN was in turn connected with high dissolution and improved solubility of APT. Furthermore, stearic acid in SLNs also influences the dissolution and absorption of APT in the gastrointestinal tract. Ren et al. carried out pharmacokinetic studies of APT on beagle dogs, obtaining average *C*_max_ and *T*_max_ values of 2413 ng/mL and 5.29 h, respectively [24].

The mean degree of absorption, determined via AUC_0–_*_t_* and AUC_0–∞_, of the APT-treated groups were 27.7 ± 4.6 and 39.2 ± 3.3 μg·h/mL, respectively, while for the groups treated with APT-CD-NP4 and APT-PX-NP8, AUC_0–_*_t_* and AUC_0–∞_ were 24.1 ± 2.1, 26.0 ± 1.6 μg·h/mL and 23.3 ± 3.4 and 25.1 ± 2.2 μg·h/mL, respectively. This shows that the APT-loaded SLN containing β-CD was more beneficial. Moreover, the oral relative bioavailability of APT, APT-CD-NP4, and APT-PX-NP8 is related to solubility and permeability of APT. APT-CD-NP4 significantly increased the oral bioavailability of the less soluble APT with low permeability through the large surface at small particle size. Palmelund et al. carried out a pharmacokinetic APT analysis on rats and obtained an AUC_0–_*_t_* value of 1198 ± 317 ng·h/mL [23]. The pharmacokinetic data suggest that APT has excellent permeability in the gastrointestinal tract and drug absorption was more rapid when APT was solubilized in polymeric material. APT-CD-NP4 was superior to APT and APT-PX-NP8 regarding oral bioavailability [25].

#### Materials and Methods

Aprepitant was obtained from Pharmasol (PVT) Ltd, Lahore, Pakistan (purity 99.91%). β-cyclodextrin and poloxamer 407 were purchased from Sigma Aldrich, Germany. Sodium lauryl sulfate (SLS) was purchased from Colorcon, Shanghai, China. Stearic acid was purchased from Lab Alley, Texas, USA. Acetonitrile, ethanol, and phosphoric acid were purchased from Merck, Germany. Double distilled water was obtained from the post-graduate research laboratory, Faculty of Pharmacy, University of Lahore.

#### Solid lipid nanoparticle preparation and solubility studies

APT and stearic acid were dissolved in different proportions (Table 6) in 10 mL ethanol (organic phase); the polymers (β-CD and poloxamer 407) were dissolved in 10 mL distilled water (aqueous phase). A suspension was obtained by adding the aqueous phase dropwise to the organic phase under continuous stirring at 1000 rpm for 1 h at 50 °C. From the suspension, the organic solvent was evaporated, and the suspension was frozen at −40 °C for 24 h and lyophilized at 1.1 mbar and −45 °C (FSF-10N-50A, China). The prepared APT-loaded SLNs were stored at 4 °C for further use [26].

**Table 6:** Composition of drug-loaded of SLNs formulations.

Formulation	Drug mass (mg)	Stearic acid mass (mg)	β-Cyclodextrin mass (mg)	Poloxamer 407 mass (mg)

APT-CD-NP1	40	40	40	—
APT-CD-NP2	40	40	80	—
APT-CD-NP3	40	40	120	—
APT-CD-NP4	40	40	160	—
APT-PX-NP5	40	40	—	40
APT-PX-NP6	40	40	—	80
APT-PX-NP7	40	40	—	120
APT-PX-NP8	40	40	—	160

The solubility of APT and SLNs was determined in 0.1 N HCl (pH 1.2) and PBS (pH 7.4). Excess amounts of APT and SLNs were added to each solvent (10 mL), mixed in a thermostatic shaker (BS-3013, China; 150 rpm) for 24 h until equilibrium was achieved at 37°C. After centrifugation at 4000 rpm for 5min, the supernatant solutions were collected through micropipette and filtered by using membrane filter (0.45 µm). The APT concentration was determined in the supernatant using a UV–vis spectrophotometer (UV 1800 Shimadzu, Japan) measuring the absorbance at λ_max_ of 210 nm [13].

#### Drug content and encapsulation efficiency

Drug content and encapsulation efficiency of APT in each formulation were determined via the total amount of drug added to the nanoparticles and the amount of free drug in the aqueous phase. Formulations equal to 2 mg of APT were dissolved in 10 mL of ethanol, filtered through a 0.45 µm membrane filter and the absorbance of the filtered solution was determined at λ_max_ = 210 nm. The experiment was performed in triplicate. The quantity of APT was measured by using a calibration curve of increasing concentrations of APT [10]. The encapsulation efficiency (EE) was calculated as follows:


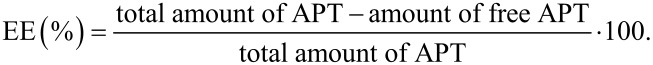


#### Physicochemical characterization

Particle size, zeta potential, and polydispersity index (PDI) of APT-NPs was analyzed through laser diffractometry (Malvern Instruments, Germany) with a measuring angle of 13° at 25 °C in triplicate [27]. Before particle size determination, APT-NPs samples were diluted with double distilled water. Scanning electron microscopy (SEM) photographs of APT-NPs were obtained on a JSM-6380A, Joel, Japan operating at a voltage of 10.0 kV. The specimens were mounted on a metallic stub with double-sided adhesive tape and gold-coated in an argon atmosphere prior to observation [28].

#### Drug excipient interaction studies

Fourier-transform infrared spectroscopy (FTIR) was performed using an Agilent Technologies Cary 660 apparatus to detect the physicochemical interaction between APT and β-CD and poloxamer 407. The spectra were recorded in a wave number range of 4000 to 400 cm^−1^. X-ray diffraction (XRD, D/MAX-2500, Rigaku, Japan) analysis was performed to evaluate the solid-state properties of APT, polymers, and APT-NPs with Cu Kα radiation. The scanning rate was 0.02°·min^−1^ in the region of 5–40° at 40 kV voltage [29]. For thermogravimetric analysis, a differential scanning calorimeter was used. About 10 mg of APT, polymers, or formulated SLNs in aluminum crucibles were heated from 10 to 500 °C at 10 K/min with nitrogen purging at 20 mL/min flow rate. The TGA cell was calibrated using tin (232 °C) and indium (156 °C) as a melting points standard.

#### In vitro drug release and kinetic modeling

In vitro release of APT from APT-NPs was evaluated using dialysis bag diffusion (*M*_W_ = 14 kDa) [30] executed in 900 mL acidic medium 0.1 N HCl (pH 1.2) with 0.1% SLS for 2 h and PBS (pH 7.4) with 0.1% SLS for 12 h using an USP dissolution apparatus-II (Curio 2020+ Lahore, Pakistan) at 100 rpm at 37 ± 0.5 °C [31]. APT-NPs (equivalent to 2 mg APT) were placed in a dialysis bag sealed at both ends in dissolution medium. After specified time intervals, samples (5 mL) were taken, replaced with fresh medium and analyzed through UV spectrophotometry at λ_max_ = 210 nm after proper dilution. The drug release kinetics were determined by applying different mathematical models including zeroth-order (Equation 1), first-order (Equation 2), Higuchi (Equation 3), and Korsmeyer–Peppas (Equation 4) models [32]:


[1] $$K_{0}=\frac{C}{t},$$

[2] $$K_{1}=\frac{2.303\log\left(C_{0}/C\right)}{t},$$

[3] $$K_{\text{H}}=\frac{Q}{\sqrt{t}},$$

[4] $$K_{\text{K}}=\frac{M_{t}}{M_{\text{a}}\cdot t^{n}},$$

where *K*_0_ is the zeroth-order rate constant, *t* is the time, *K*_1_ is the first-order rate constant, *C*_0_ is the initial concentration of the drug, and *K*_H_ is the Higuchi model rate constant. *K*_K_ is the Korsmeyer–Peppas model or drug–polymer kinetic constant, *M_t_*/M_a_ is the fraction of drug release at time *t*, and *n* is the exponent of drug release mechanism.

#### Statistical analysis

Interpretation of in vitro data was performed using ANOVA. Dunnett’s multiple comparison tests were employed by Graph Pad Prism 8.0.2 software (Graph Pad Software, Inc. San Diego, USA) to evaluate statistically significant differences and level of significance was fixed at 95% (*p* < 0.05) [16].

#### Stability studies

Accelerated stability studies were performed to check whether APT would revert to the crystalline form from the SLN formulation. The optimal formulation APT-CD-NP4 (240 mg) was added to a glass vial and stored in a stability chamber (Hi-Tech, India) at 40 °C/75% RH for six months in total. PXRD studies were performed to analyze samples for any formulation change after one, two, three, and six months [33].

### In vivo pharmacokinetic studies

#### Study protocol

10 male rabbits (2 kg body weight) were housed in an animal house under standard environmental conditions of 22 ± 3 °C and 45% relative humidity under a 12 h dark/light cycle (Faculty of Pharmacy, University of Lahore, Pakistan). The Institutional Research Ethical Committee (IREC) at University of Lahore approved the study protocol, Lahore vide no. IREC-2021-50. Rabbits were permitted to water and fasted for 12 h before treatment. The rabbits were divided into three distinct treatment groups (*n* = 3), APT and APT-NPs (5.513 mg/kg of APT) were given orally.

#### Blood sampling and chromatography

Blood samples (1 mL) were obtained using a catheter embedded into the jugular vein of the rabbits at 0.5, 1, 2, 4, 6, 12, and 24 h after dosing, gathered in heparinized tubes, and put on wet ice until centrifugation. Plasma was centrifuged at 2500 rpm for 15 min. Acetonitrile (1 mL) was added to plasma samples to precipitate proteins. Samples were vortexed for 30 s and centrifuged for 10 min at 13,500 rpm. The sampling procedure was similar to that of Erdoğar and colleagues [16]. The supernatant was isolated, and the drug content was determined via high-performance liquid chromatography at λ_max_ = 210 nm. Analysis was performed by utilizing a C18 column (4.6 × 250 mm, 5 μm, Shim-pack Substance, Japan) at a flow rate of 1.5 mL/min and at 40 °C. Tests were performed utilizing a combination of 0.1% phosphoric acid in acetonitrile (55:45 v/v) as mobile phase. The sample injection volume was 100 μL, and the run time was 0.1 mL/min. A linear regression plot was constructed by the least square method , and was used to estimate the amount of APT in rabbit plasma.

#### Pharmacokinetic parameters

The following non-compartmental parameters were calculated by using PK Solver: half-life (*t*_1/2_), time to reach maximum concentration of drug in plasma (*T*_max_), maximum concentration of drug in plasma (*C*_max_), mean residence time (MRT), and area under the plasma concentration curve (AUC).

## Conclusion

APT-loaded SLN formulations with the polymers β-cyclodextrin and poloxamer 407 were successfully prepared and characterized in this study. The use of β-cyclodextrin and poloxamer 407 in SLNs significantly increased the solubility and dissolution rate of APT. Particle size, zeta potential, and drug content of the SLN formulation APT-CD-NP4 were 121.1 nm, −18.8 mV, and 96.75%, respectively. SEM analysis confirmed the smooth surface morphology of SLN formulations. FTIR, DSC, SEM, and XRD suggested that there was no physical interaction between APT and the polymers. Moreover, β-CD-based formulation showed better in vitro release behavior than the poloxamer 407-based formulations in phosphate-buffered saline (pH 7.4) and 0.1 N HCl (pH 1.2). The optimal SLN formulation APT-CD-NP4 is a promising tool for oral sustained-release dosage in order to improve the bioavailability of the BCS class-IV drug APT.

## Data Availability

The data generated during and analyzed during the current study are available from the corresponding author upon reasonable request.
